# Geographic Co-distribution of Influenza Virus Subtypes H7N9 and H5N1 in Humans, China

**DOI:** 10.3201/eid1911.130815

**Published:** 2013-11

**Authors:** Liya Wang, Wenyi Zhang, Ricardo J. Soares Magalhaes, Archie C.A. Clements, Wenbiao Hu, Fan Ding, Hailong Sun, Shenlong Li, Qiyong Liu, Zeliang Chen, Yansong Sun, Liuyu Huang, Cheng-Yi Li

**Affiliations:** Institute of Disease Control and Prevention of People’s Liberation Army, Beijing, China (L. Wang, W. Zhang, H. Sun, S. Li, Z. Chen, Y. Sun, L. Huang, C.-Y. Li);; University of Queensland, Brisbane, Queensland, Australia (R.J. Soares Magalhaes, A.C.A. Clements, W. Hu); and Chinese Center for Disease Control and Prevention, Beijing (F. Ding, Q. Liu)

**Keywords:** geographic co-distribution, influenza, subtypes, H7N9, H5N1, humans, China, avian influenza, influenza A, viruses, low pathogenicity, highly pathogenic

**To the Editor:** Human infection with a novel low pathogenicity influenza A(H7N9) virus in eastern China has recently raised global public health concerns (*1*). The geographic sources of infection have yet to be fully clarified, and confirmed human cases from 1 province have not been linked to those from other provinces. While some studies have identified epidemiologic characteristics of subtype H7N9 cases and clinical differences between these cases and cases of highly pathogenic influenza A(H5N1), another avian influenza affecting parts of China (*2*–*4*), the spatial epidemiology of human infection with influenza subtypes H7N9 and H5N1 in China has yet to be elucidated. To test the hypothesis of co-distribution of high-risk clusters of both types of infection, we used all available data on human cases in mainland China and investigated the geospatial epidemiologic features.

Data on individual confirmed human cases of influenza (H7N9) from February 19, 2013, through May 17, 2013, and of influenza (H5N1) from October 14, 2005, through May 17, 2013, were collected from the Chinese Center for Disease Control and Prevention. The definitions of these cases have been described (*3*,*5*). A total of 129 confirmed cases of influenza (H7N9) (male:female ratio 2.39:1) and 40 confirmed cases of influenza (H5N1) (male:female ratio 0.90:1) were included in the analysis. The median age of persons with influenza (H7N9) was higher than for persons with influenza (H5N1) (58 years vs. 27 years; z = −7.73; p<0.01). Most (75.0%) persons with influenza (H5N1) had direct contact (e.g., occupational contact) with poultry (including dead and live birds) or their excrement and urine, whereas most (64.3%) persons with influenza (H7N9) had only indirect exposure to live poultry, mainly during visits to live poultry markets.

Reported cases of influenza (H5N1) were distributed over 40 townships in 16 provinces, whereas cases of influenza (H7N9) were relatively more concentrated, in 108 townships but only 10 provinces (Figure). To identify a spatial overlap between the primary cluster of influenza (H7N9) cases, detected in April 2013 (relative risk [RR] 78.40; p<0.01), and the earliest space-time cluster of influenza (H5N1) cases, detected during November 2005–February 2006 (RR 65.27; p<0.01), we used spatiotemporal scan statistics with a maximum spatial cluster size of 5% of the population at risk in the spatial window and a maximum temporal cluster size of 25% of the study period in the temporal window (*6*) (Figure). The results suggest that the overlap is not perfect and is concentrated around an area southeast of Taihu Lake (south of Jiangsu Province), bordering the provinces of Anhui and Zhejiang. Smaller clusters of influenza (H7N9) cases were identified in the boundary of Jiangsu and Anhui Province (8 cases; RR 64.86; p<0.01) and Jiangxi Province (Nanchang County and Qingshanhu District) (4 cases; RR 105.67; p<0.01). A small cluster of influenza (H5N1) cases was detected during 2012–2013 along the boundaries of Guanshanhu, Yunyan, and Nanming Counties in Guizhou Province (3 cases; RR 496.60; p<0.01). 

**Figure:**
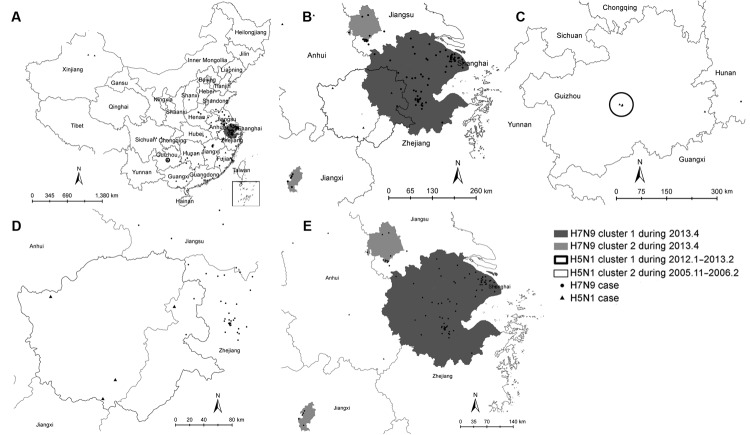
Geographic and temporal distribution of human cases of infection with avian influenza subtypes H7N9 (circles) and H5N1 (triangles), China. A) Distribution and space-time clusters of human influenza (H7N9) and influenza (H5N1) cases, calculated by using Kulldorff’s scan statistics in SaTScan version 9.1.1 (*6*). B) Spatial overlap between influenza (H7N9) and influenza (H5N1) case clusters in an area bordering the provinces of Anhui and Zhejiang. C) Primary cluster of influenza (H5N1) cases in Guizhou Province (relative risk [RR] 496.60). D) Secondary cluster of influenza (H5N1) cases in Anhui and Zhejiang Provinces (RR 65.27). E) Primary (RR 78.40) and secondary clusters of influenza (H7N9) cases on the boundary of Jiangsu and Anhui Provinces (RR 64.86) and in Jiangxi Province (RR 105.67).

In addition, family clustering, defined as >2 family members with laboratory-confirmed cases, was found for influenza (H7N9) cases during March–April 2013 in Shanghai and Jiangsu Provinces and for influenza (H5N1) cases during December 2007 in Jiangsu Province. Family clustering may indicate person-to-person viral transmission or may reflect common exposure to infected poultry or their excrement in the household or in a contaminated environment (*7*). No evidence supports person-to-person viral transmission as the means of transmission in family clusters.

In conclusion, we found compelling evidence that the high-risk areas for human infection with subtype H7N9 and H5N1 viruses are co-distributed in an area bordering the provinces of Anhui and Zhejiang, which suggests that this area might be a common ground for the transmission of emerging avian influenza viruses in China. We also found that visits to live poultry markets or exposure to contaminated environments are a pathway to infection with influenza (H7N9) virus, whereas infection with influenza (H5N1) is more tied to occupational hazards. These differences may reflect the differences in the pathogenicity of the viruses in poultry, which influences disease progression and identification of clinical signs further down the poultry market chain. Further empirical investigation into our findings could identify risk factors that might be involved in disease transmission to humans in high-risk areas and could help public health authorities develop targeted control and surveillance strategies to prevent disease transmission.
